# Circadian Rhythms, Chrononutrition, Physical Training, and Redox Homeostasis—Molecular Mechanisms in Human Health

**DOI:** 10.3390/cells13020138

**Published:** 2024-01-11

**Authors:** Cristina Manuela Drăgoi, Alina Crenguţa Nicolae, Anca Ungurianu, Denisa Marilena Margină, Daniela Grădinaru, Ion-Bogdan Dumitrescu

**Affiliations:** 1 Department of Biochemistry, Faculty of Pharmacy, Carol Davila University of Medicine and Pharmacy, 020956 Bucharest, Romania; cristina.dragoi@umfcd.ro (C.M.D.); alina.nicolae@umfcd.ro (A.C.N.); anca.ungurianu@umfcd.ro (A.U.); denisa.margina@umfcd.ro (D.M.M.); 2 Department of Physics and Informatics, Faculty of Pharmacy, Carol Davila University of Medicine and Pharmacy, 020956 Bucharest, Romania; ion.dumitrescu@umfcd.ro

**Keywords:** circadian rhythm, oxidative stress, physical exercise, chronobiology, metabolism, redox homeostasis, chronotype, sport performance, sleep

## Abstract

A multitude of physiological processes, human behavioral patterns, and social interactions are intricately governed by the complex interplay between external circumstances and endogenous circadian rhythms. This multidimensional regulatory framework is susceptible to disruptions, and in contemporary society, there is a prevalent occurrence of misalignments between the circadian system and environmental cues, a phenomenon frequently associated with adverse health consequences. The onset of most prevalent current chronic diseases is intimately connected with alterations in human lifestyle practices under various facets, including the following: reduced physical activity, the exposure to artificial light, also acknowledged as light pollution, sedentary behavior coupled with consuming energy-dense nutriments, irregular eating frameworks, disruptions in sleep patterns (inadequate quality and duration), engagement in shift work, and the phenomenon known as social jetlag. The rapid evolution of contemporary life and domestic routines has significantly outpaced the rate of genetic adaptation. Consequently, the underlying circadian rhythms are exposed to multiple shifts, thereby elevating the susceptibility to disease predisposition. This comprehensive review endeavors to synthesize existing empirical evidence that substantiates the conceptual integration of the circadian clock, biochemical molecular homeostasis, oxidative stress, and the stimuli imparted by physical exercise, sleep, and nutrition.

## 1. Introduction

Scientific studies have firmly established the indispensable role of the circadian system in orchestrating key biological processes, encompassing crucial aspects such as homeostasis, cardiovascular regulation, and glucose metabolism. Disruptions to the circadian clock, characterized by disturbances or the loss of rhythmicity, have emerged as prominent contributors to the development of various diseases and premature aging, consequently justifying recognition as significant health-related concerns [1,2,3,4,5,6,7].

Within the realm of physiological regulation, redox reactions involving reactive oxygen and nitrogen species hold control, governing intricate processes including cellular signaling and immune responses. However, the pathological repercussions of oxidative stress manifest as disruptions in cellular signaling and deleterious modifications to pivotal biomolecules, deoxyribonucleic acid (DNA), proteins, and lipids [8,9]. Notably, a bi-directional relationship has been established between circadian rhythms and oxidative stress. The perturbation of circadian rhythms can exert profound influence on redox biology, while alterations in redox homeostasis can reciprocally impact the regulation of circadian processes. For instance, key antioxidant enzymes, including superoxide dismutase (SOD), glutathione peroxidase (GSH-Px), and catalase (CAT), exhibit obvious circadian patterns in their expression and activity [1,10,11,12,13].

This interplay between circadian rhythmicity and redox biology has unfolded as a captivating frontier within the grounds of human health. The impact of physical exercise, as a potent environmental signal, extends beyond its recognized health benefits, involving also the capacity to rectify perturbations in circadian systems. It is noteworthy that the response to an exercise stimulus may also manifest circadian variability. Concurrently, inquiries into the convoluted relationship between exercise, reactive species, and oxidative stress have unveiled that oxidative species generated through exercise can yield both beneficial and potentially deleterious health outcomes, reliant upon the specific nature, intensity, and duration of the exercise regimen [3,14,15].

Remarkably, the emerging link between circadian rhythmicity and redox metabolism remains a new territory concerning its intersection with exercise, nutrition, and sleep.

## 2. Circadian Rhythms in the Regulation of Human Physiological and Behavioral Processes

Biological rhythms encompass predictable patterns of physiological and behavioral changes occurring in living organisms. These endogenous rhythms can persist independently of external cues, although they continually interact with and adapt to environmental fluctuations. Humans exhibit a complex network of biological signaling and communication, involving systems like the immune system, gastrointestinal tract, and central nervous system, which operate in coordinated rhythmic patterns. These biological rhythms play a crucial role in maintaining the overall homeostasis, ensuring the organism’s balance and stability. These rhythms are categorized based on their cycle durations into three types: circadian rhythms (about 24 h), ultradian rhythms (much shorter than 24 h, often lasting seconds to minutes), and infradian rhythms (longer than 24 h) [16,17].

The body’s central biological clock, the suprachiasmatic nucleus (SCN) located in the hypothalamus, houses nerve cells that govern most circadian rhythms in mammals. The SCN functions as the “master biological clock”, generating circadian rhythms through a negative feedback loop involving clock genes. For synchronizing this central pacemaker with the 24 h day-night cycle, the entrainment process and the external signals known as *zeitgebers* are essential. The most influential environmental cue for entrainment is light, with the retinohypothalamic tract serving as the primary pathway for photic entrainment [10,18,19].

Measuring circadian physiology involves overcoming challenges posed by external factors like sleep-wake cycles and internal circadian rhythms. The suprachiasmatic nucleus, responsible for regulating the circadian pacemaker, can be affected by numerous external and internal factors, including light exposure, temperature, dietary patterns, activities, and stress levels, complicating the isolation of its contributions to observed rhythmic events [18].

Melatonin serves as a commonly used marker for measuring circadian phase, as its levels are less influenced by biochemical and physiological variables. Melatonin levels in the blood typically rise a few hours before the onset of sleepiness, preceding nocturnal sleep in healthy individuals. The initiation of this increase is known as the dim light melatonin onset (DLMO), and saliva is often sampled to measure it because it strongly correlates with plasma melatonin levels. To obtain precise measurements, subjects must remain in low light conditions, avoid physical activity and certain food products before saliva collection, and, depending on the study’s design, collect saliva samples hourly over a 24 h period to construct a partial melatonin curve [20,21,22].

### 2.1. The Circadian Clock and the Sleep Homeostasis

The interaction between the circadian clock and the sleep homeostasis is described by the “two-process model”, illustrating how these systems regulate sleep. Sleep is governed by two physiological processes: process S, the homeostatic process, and process C, the circadian process, which compete to regulate wakefulness and sleep patterns. The homeostatic process, corresponding to hunger or thirst, accumulates sleep pressure during wakefulness and dissipates it during sleep. Adenosine, a metabolic byproduct of cellular activity, accumulates in the brain during wakefulness and stimulates adenosine receptors, contributing to increased sleepiness and decreased alertness. Conversely, during sleep, adenosine is cleared, leading to decreased adenosine receptor stimulation and increased alertness [14,23].

The circadian process, process C, regulates the 24 h rhythm of sleep and wakefulness and is primarily influenced by melatonin, which responds strongly to light-dark exposure but can also be modulated by other *zeitgebers* such as food or physical activity. The quality and timing of sleep within a 24 h cycle depend on the interaction between processes C and S: when process S is high and process C is low, the probability and quality of sleep are improved. However, disorders like depression or sleep phase disorders can lead to a misalignment of these processes, resulting in insomnia or non-restorative sleep. Additionally, caffeine, an adenosine receptor antagonist, can temporarily promote alertness, but chronic use may lead to receptor upregulation, reducing its effectiveness and potentially necessitating periodic interruptions to maintain its wake-promoting effects [24,25].

### 2.2. The Influence of Circadian Rhythms on Human Cognitive and Physical Performance

The daily rhythm represents a prominent contributor to physical and cognitive performance variability, with the magnitude of this variability increasing as task complexity rises. This circadian performance rhythm displays oscillations ranging from 10% to 30% of the daily average, highlighting the significance of exploring the timing of athletic performance with meticulous attention. Research findings indicate that daily rhythmic oscillations are evident in various physiological and behavioral functions that impact athletic performance, encompassing baseline levels of sensory-motor, psycho-motor, and perceptual factors. Furthermore, studies suggest that factors such as workload, physiological stressors, motivation, arousal levels, chronotype, environmental lighting, sleep patterns, and the “post-lunch dip” phenomenon can modulate rhythmicity in components of athletic performance.

The circadian rhythm has been acknowledged to have a significant influence on human cognitive and physical functioning, through a series of human studies [26,27,28,29]. Specifically, a 20 h forced desynchrony protocol study revealed a circadian pattern of performance in tests of psychomotor vigilance, short-term memory, calculation, digit symbol substitution, and alertness, with peak performance occurring close to the maximum core body temperature (CBT), just before melatonin secretion began. On the other hand, a significant decline in performance was observed around the minimum CBT, just after the peak of melatonin production. Additionally, after adjusting for circadian effects, cognitive performance scores decreased with longer periods of wakefulness [28,30].

Early chronobiological studies assessed the diurnal fluctuation in various performance-related abilities, such as elbow flexion strength, simple reaction time, maximal information processing rate, physiological tremor, and eye-hand tracking capacity [31]. The results obtained by Freivalds et al. demonstrated that performance ratings were generally higher during the day and evening than at night or in the morning, despite modest amplitudes of fluctuation. Similarly, the study conducted by Teo et al. examined the circadian cycle of cortisol and testosterone in relation to strength and power performances at four different time intervals throughout the day [32]. The study revealed that peak power output varied with time, reaching the highest level at 16:00, with no association between blood levels of cortisol or testosterone and power output [32].

Although the mentioned studies had limitations, such as not using a constant routine or forced desynchrony technique and having limited measurements, the research supports the idea of better cognitive and physical performance in the late afternoon than in the morning, potentially due to the influence of the circadian pacemaker.

### 2.3. The Link between Circadian Rhythms and Exercise in Terms of Performance and Health Benefits

More recent studies bring new and useful data regarding the correlations between physical training, circadian alignment, and biological signals emerging from sport activities in the human organism. Hence, the circadian clock can be synchronized by both photic and non-photic stimuli, as temperature, physical activity, and food intake. In light of the fact that exercise represents a significant challenge to the body’s overall equilibrium, leading to widespread disruptions at the cellular, tissue, and organ levels, modern theories have started exploring the connections between exercise and circadian rhythms in terms of performance and health benefits [33,34,35,36,37]. A substantial body of research has demonstrated that exercise can influence the circadian system in rodents, and expanding evidence in humans suggests that exercise can induce phase-shifting effects, potentially influenced by an individual’s chronotype [38,39,40]. Exercise appears to be a potent factor for entraining both central and peripheral clocks, including those within skeletal muscle. For example, male rugby players exhibit significantly higher average expression of core-clock genes compared to sedentary males [41].

Accordingly, researchers and clinicians are increasingly drawn to the possibility that exercise could mitigate the adverse health effects of circadian misalignment and that there might be an optimal time for exercise to maximize its therapeutic benefits. Among all peripheral tissues, skeletal muscle stands out as a major downstream target for circadian clock activity.

Molecular clocks intricately regulate the transcriptional activity of numerous clock-controlled genes. This regulation occurs through direct actions involving master heterodimeric transcription factors CLOCK and BMAL1, alongside other clock-regulated transcription factors, or indirectly via various clock output proteins. This orchestration establishes rhythmic gene expression patterns, thus regulating diverse biological functions under circadian control. The heterodimer composed of CLOCK and BMAL1 also rhythmically initiates the expression of their transcriptional repressors, Period (Per1 and Per2) and Cryptochrome (Cry1 and Cry2) [13,14,42,43,44].

Studies have shown that the molecular clock plays a crucial role in regulating glucose metabolism in skeletal muscle [3,45,46]. For instance, deletion of BMAL-1 in mouse skeletal muscle leads to impaired glucose uptake, reduced levels of GLUT-4, and disruption of key glycolytic enzymes. Consequently, disruptions in the molecular clock within muscle tissue appear to be relevant to the development of metabolic conditions like type 2 diabetes. Given that exercise is recommended for the prevention and treatment of type 2 diabetes, it seems that the metabolic benefits of exercise are, at least in part, achieved through its effects on the molecular clock in muscle, thereby restoring local circadian regulation. This is exemplified by increased gene and protein expression of BMAL-1 and PER2 in skeletal muscle of adults with obesity and pre-diabetes after 12 weeks of exercise training, which coincided with improvements in body composition, peripheral insulin sensitivity, and maximal oxygen consumption. Specifically, BMAL-1 gene expression was positively correlated with glucose disposal rate [41,47].

While exercise undeniably has favorable effects on skeletal muscle metabolism, relatively little is known about the potency of these effects at different times of the day. This is significant because the biological clock appears to drive discernible rhythms in human skeletal muscle metabolism. Mitochondrial oxidative capacity, a critical determinant of exercise performance, follows a daily rhythm, peaking in the late evening and reaching its lowest point in the early afternoon. As oxidative capacity plays a vital role in exercise performance, it is not surprising that studies report strong time-of-day effects on exercise performance, capacity, and strength measures in humans, with some of these variations correlating with daily profiles of the PER2 gene [45]. These performance fluctuations are thought to involve circadian fluctuations in core temperature, endocrine hormones, neuromuscular function, and metabolic flux. Body temperature, a key contributing factor to these performance fluctuations, tends to peak in the late afternoon, and as exercise-related thermoregulation also follows a circadian rhythm, it may explain differences in the onset of fatigue when the same activity is performed at different times of the day, especially in longer endurance-type exercise sessions. Variations in exercise efficiency in humans, such as improved efficiency in the late afternoon compared to the early morning, have been attributed to clock-driven fluctuations in metabolic control, particularly in carbohydrate metabolism, which requires less oxygen consumption, lower heart rate, and lower perceived exertion. In competitive and elite sports contexts, this connection between exercise capacity and the molecular clock may be relevant when planning optimal training and competition schedules, although it is not a completely new concept [14,28,37,48].

Aligning exercise sessions, along with other chronobiological measures such as time-restricted eating, optimal sleeping patterns, and chronotype assessment, presents an attractive paradigm for maximizing the metabolic and health benefits of exercise.

### 2.4. The Circadian Regulation of Glucose Metabolism

In the context of glucose metabolism, factors like insulin and cortisol exhibit expression and secretion patterns synchronized with circadian cues, similar to the main organs involved in glucose uptake and metabolism (e.g., liver, pancreas, adipose tissue, muscles). Glucose tolerance reaches its peak during daylight hours and declines during the nocturnal period. Insulin production follows a temporal pattern influenced by both feeding-fasting cycles and circadian rhythms. Nutrient levels in the bloodstream act as signals for insulin production, modulated also by the circadian and suprachiasmatic nucleus (SCN) systems [43,49,50].

Pancreatic β-cells receive parasympathetic input, regulated by GABA-ergic projections from the SCN, while the SCN influences the liver through glutamatergic and GABA-ergic projections, modulating glucose production. The timing of peak functionality in pancreatic β-cells remains uncertain, though some data suggest it may occur during midday, indicating enhanced postprandial glucose regulation with carbohydrate intake at this time. Cortisol, a steroid hormone essential to metabolism and stress responses, also exhibits daily rhythmicity, with peak levels coinciding with the start of the active phase.

Circadian clocks respond to environmental cues, with light being the most potent signal for the SCN clock system. Food availability and patterns of activity and rest serve as crucial *zeitgebers* for peripheral clocks governing local physiological processes. Synchronization of peripheral clocks is essential for coordinated physiology. Disruptions in circadian rhythms can lead to disturbances in metabolism, resulting in various adverse consequences, from irritability and fatigue to chronic diseases like obesity, type 2 diabetes mellitus, cardiovascular diseases, and inflammation [10,12,43,51,52,53,54,55,56,57].

### 2.5. The Entrainment Effect of Exercise on Circadian Rhythms: Experimental and Clinical Proofs

Exercise serves as a non-photic stimulus that can influence the entrainment of circadian clocks. In studies conducted in rodents kept in constant dark conditions, it has been observed that exercise can shift the phase of circadian rhythms related to wheel running behavior. For instance, there were reported changes in the expression of Per1 and Per2 genes in the suprachiasmatic nucleus in response to wheel running during constant darkness. Furthermore, the timing of exercise also plays a role in regulating circadian clocks. When wheel running occurs at the beginning of the active phase, it has a more significant impact on reducing the amplitude of Per2 gene expression in the SCN compared to exercise taking place at the end of the active phase. Scheduled exercise has the ability to entrain the molecular clocks in peripheral tissues like skeletal muscle and lungs, although not the SCN, especially when conducted under light-dark conditions. In addition, scheduled exercise-induced entrainment of Per2 gene expression was demonstrated in the submandibular gland, suggesting that programmed exercise can synchronize the molecular clock in both the SCN and peripheral tissues, but its ability to entrain the master clock is rather limited in the absence of light [20,21,58,59,60].

Similar to observations in mice and rats, exercise-induced phase shifts of circadian rhythms have also been documented in humans, concluding that exercise accelerated phase delays in circadian rhythms induced by forced sleep in humans. The daily rhythm of plasma melatonin levels was used as an indicator of circadian rhythms, and bicycle ergometer exercise under dim light conditions facilitated the 9 h sleep-schedule-induced phase delay of the circadian melatonin rhythm [20]. Additionally, there were reported differential effects of exercise on the circadian melatonin rhythm and the sleep-wake cycle in humans. Exercise appeared to expedite the re-entrainment of the sleep-wake cycle, particularly under dim light conditions and a restricted phase-advanced sleep schedule. However, exercise did not have the same impact on the melatonin rhythm under these conditions. In another study which investigated the effects of exercise on the circadian melatonin rhythm and sleep-wake cycle under bright light and an 8 h phase-advance shifted sleep-wake schedule, it was found that the sleep-wake cycle was entrained by the sleep schedule regardless of exercise, but exercise played a role in advancing the circadian rhythm of melatonin. Thus, the combination of light exposure and exercise emerges as a potent entrainment cue for circadian rhythms in humans [61,62].

## 3. Chrononutrition: The Connection between Circadian Rhythms, Nutrients, and the Timing of Food Intake

Recently, there has been an increase in attention regarding a much more multifaceted concept that has appeared to delineate the interplay between dietary factors and the circadian system, namely chrononutrition. The term “chrononutrition” encompasses the problematic relationship between the timing of dietary intake, meal composition, and the body’s daily circadian rhythms, all of which profoundly affect metabolic health. This concept emphasizes that, in addition to the quantity and composition of food, the timing of food intake plays a critical role in an organism’s well-being. It suggests an “optimal” feeding schedule aligned with the body’s metabolic rhythms to promote general health [42].

Unlike variations in daylight patterns dictated by geographical location, changes in food intake and feeding timing significantly impact nutrient-sensing pathways that contribute to homeostasis. Synchronizing food consumption, food quality and quantity, and metabolic rhythms throughout the day may optimize metabolism and promote overall health. Several neurotransmitters play pivotal roles in regulating the sleep-wake cycle, encompassing 5-hydroxytryptophan, serotonin, melatonin, gamma-aminobutyric acid (GABA), orexin, acetylcholine, galanin, noradrenaline, and histamine [63]. Consequently, nutritional interventions targeting these neurotransmitter systems have the potential to exert a favorable influence on sleep patterns. Dietary precursors hold the capacity to impact the rate of synthesis and functionality of neurotransmitters. For example, the synthesis of serotonin, a neurotransmitter, is contingent upon the availability of its precursor, tryptophan, within the brain. Tryptophan is transported across the blood–brain barrier via a system that shares transporters with various large neutral amino acids (LNAA). The ratio of tryptophan to LNAA in the bloodstream is critical for the transport of tryptophan into the brain and can be augmented through the consumption of tryptophan-rich foods, adherence to a high-carbohydrate/low-protein diet, or the ingestion of α-lactalbumin, a protein derived from whey [64,65].

### 3.1. The Bidirectional Relationships among Tryptophan, Serotonin (5-HT), Physical Activity, Sleep, and Dietary Patterns

**Figure 1:**
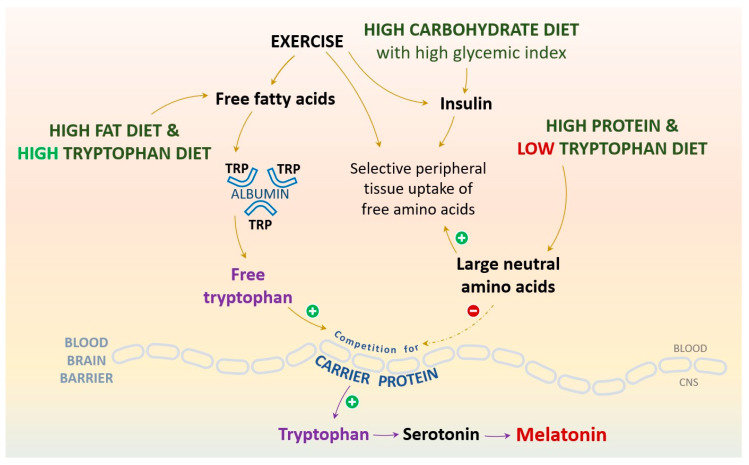
The increase in melatonin production can be achieved through various nutritional interventions that aim to enhance tryptophan (Trp) availability at the CNS level. Generally, this process can be realized by either increasing free Trp availability or reducing the relative plasma concentration of large neutral amino acids (LNAA). The passage of tryptophan through the blood–brain barrier constitutes a multifaceted process, as the active transport of amino acids to the brain, performed by a particular carrier protein, is accessible to all LNAA and is not exclusive to tryptophan. So, tryptophan must engage in competition with other LNAA, which are often more abundantly available in the food supply, to secure transport into the brain. To augment the availability of tryptophan for serotonin and melatonin synthesis, it is advantageous to redirect these competing amino acids toward peripheral tissues. This diversion can be facilitated through the release of insulin, which in turn fosters protein synthesis in muscle tissue. Notably, the process of insulin shunting effectively diminishes the pool of LNAA that reach the brain, thereby liberating transporters for tryptophan binding. In light of this mechanism, the co-consumption of carbohydrates in conjunction with tryptophan-rich foods holds the potential to enhance the entry of tryptophan into the brain. These objectives can also be accomplished by adopting a high-protein diet richer in tryptophan compared to LNAA, consuming carbohydrates to elevate the free Trp-to-branched-chain amino acid (BCAA) ratio and stimulate insulin release, which facilitates BCAA uptake into muscle. Additionally, melatonin production can be influenced by the consumption of high-fat meals, leading to increased free fatty acids and subsequently higher free Trp levels. Furthermore, engaging in physical exercise can impact both free fatty acids and insulin levels, contributing to the intensification of melatonin synthesis.

The neurotransmitter 5-HT assumes a pivotal role in the regulation of a myriad of physiological functions encompassing mood regulation, sleep configuration, pain perception, and the orchestration of food intake timing and patterns. In the context of physical activity, 5-HT is implicated in the progression of central fatigue through diverse biochemical pathways. The biosynthesis of 5-HT is depending upon the availability of the essential amino acid tryptophan, with cortisol emerging as a critical regulator of tryptophan hydroxylase, a crucial synthetic enzyme [66]. Unlike the synthesis of noradrenaline and dopamine, which is not affected by changes in tyrosine levels, tryptophan levels have a direct impact on 5-HT synthesis. This is due to the enzyme’s unsaturated state with its substrate, tryptophan. Elevated plasma levels of free tryptophan may increase 5-HT concentrations in neurons. Studies have shown that increased levels of free tryptophan can increase 5-HT concentrations by 35% in the brains of exercising rats. The translocation of tryptophan across the blood–brain barrier is facilitated by the carrier protein for large neutral amino acids (LNAA) [64] (Figure 1).

The nutritional perspective states that the intake of large neutral amino acids, especially tyrosine and the branched-chain amino acids (BCAA), alters tryptophan transport into the brain and its conversion to serotonin and melatonin. The passage of tryptophan through the blood–brain barrier constitutes a multifaceted process, as the active transport of amino acids to the brain is accessible to all LNAA and is not exclusive to tryptophan. To augment the availability of tryptophan for serotonin and melatonin synthesis, it is advantageous to redirect these competing amino acids toward peripheral tissues. This diversion can be facilitated through the release of insulin, which in turn fosters protein synthesis in muscle tissue. In light of this mechanism, the co-consumption of carbohydrates in conjunction with tryptophan-rich foods holds the potential to enhance the entry of tryptophan into the brain. Consequently, this dietary strategy can facilitate the biosynthesis of serotonin and melatonin. Manipulating blood LNAA levels through the consumption of certain nutritive sources can induce significant disparities in brain tryptophan availability and serotonin and melatonin synthesis rates, thereby generating predictable outcomes concerning mood, cognitive function, sleep-wake cycles, and hormone secretion, primarily involving prolactin and cortisol [25,67,68].

The consumption of mixtures of LNAA, particularly BCAA, decreases brain tryptophan transport rate and serotonin synthesis yield. Although this is projected to improve physical performance by lowering serotonin availability, such effects are generally considered modest. Nevertheless, it is noteworthy that BCAA consumption also decreases tyrosine transport and dopamine synthesis in the brain. Possibly, BCAA combined with tyrosine could prevent the decline in dopamine, while still causing a decline in the serotonin level [68].

The physical activity perspective reveals that BCAA, which are required by active muscles during physical activity, decrease competition for tryptophan in the blood–brain barrier, leading to increased tryptophan penetration into the brain. During physical activity, the lipolytic effect mobilizes more free fatty acids in the plasma, which displaces albumin-bound tryptophan, increasing even further the amount of free tryptophan available to the brain. Both of these processes may contribute to central fatigue during exercise. Fatigue could also be accounted for in terms of energy: although this is unlikely for glucose, slightly lower levels of either glucose or oxygen may also have an impact on brain metabolism during physical activity. Supplementation of tryptophan has been shown to have a cognitive benefit, but has not consistently been shown to reduce fatigue during physical activity [14,67,68].

### 3.2. Melatonin, the Modulator of Cellular Redox Homeostasis, Human Circadian Rhythms, and Sleep-Wake Cycle

**Figure 2:**
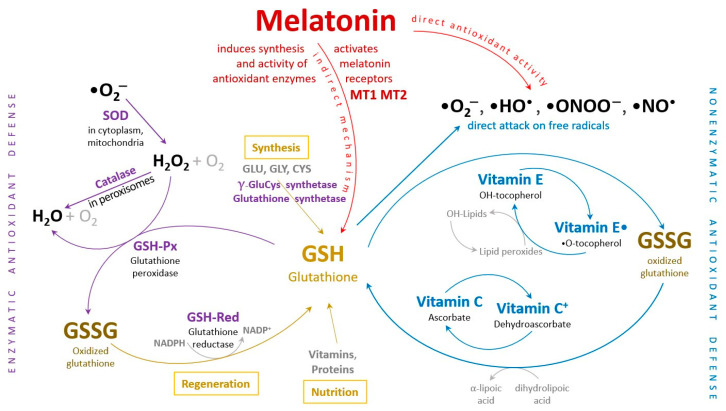
The human organism’s antioxidant defense mechanism involves a cooperative interaction between enzymatic and non-enzymatic antioxidant systems to collectively shield the cells and organ systems from harm caused by free radical damage. *ROS—reactive oxygen species, SOD—Superoxide dismutase, GSH—reduced glutathione, GSSG—oxidized glutathione, GSH-Px—Glutathione peroxidase, GSH-R—Glutathione reductase.*

Melatonin, chemically referred to as N-acetyl-5-methoxytryptamine, is an endogenous indoleamine that plays a crucial role in regulating various physiological processes. These processes include the human circadian rhythms, sleep-wake cycle, anxiety, immune response, and cardiac function. Melatonin also influences appetite and helps regulate insulin levels, among other functions. Importantly, both melatonin and its metabolites are potent antioxidants, effectively scavenging reactive oxygen species (ROS) and reactive nitrogen species (RNS) to protect mitochondria from oxidative damage. Melatonin and its derivatives additionally stimulate the activity of several antioxidant enzymes, maintaining the cellular redox homeostasis and playing a significant role in the aging process [49,69] (Figure 2).

Disruption of cellular redox balance can be triggered, in certain circumstances, by intense physical activity, similarly encountered in unhealthy lifestyle behaviors such as smoking, alcohol consumption, unbalanced diet, and exposure to environmental factors like radiation, viruses, and bacteria. Intense and prolonged exercise can lead to inflammation due to the high generation of free radicals, ROS/RNS, and potential oxidative damage to muscle tissues. Conversely, regular moderate-to-vigorous exercise results in a moderate concentration of ROS/RNS, triggering adaptive responses that benefit the organism and contribute to the prevention and management of diseases associated with ROS/RNS. In the context of sports, maintaining the redox state in skeletal muscle is particularly critical, as it depends on the efficiency of ROS generation [14,70,71,72].

Changing the perspective, emerging evidence indicates that physical exercise can have both immediate and delayed effects, occurring within 12 to 24 h, on the secretion of melatonin in humans. The precise mechanism through which reduced sleep impacts glucose metabolism is believed to be complex, involving various factors such as diminished brain glucose utilization, shifts in sympatho-vagal balance, elevated evening cortisol levels, prolonged nighttime growth hormone secretion, and the initiation of pro-inflammatory processes [73,74,75].

Leptin and ghrelin are pivotal hormonal regulators of food intake, with leptin exerting inhibitory effects on food consumption and ghrelin acting as an appetite-stimulating hormone. Numerous studies have demonstrated that sleep deprivation results in decreased leptin levels and increased ghrelin levels, leading to sleep-restriction-induced heightened hunger and appetite, particularly for carbohydrate-rich foods [76,77,78,79,80,81,82]. Furthermore, beyond the alterations in appetite-regulating hormones, the functioning of two major neuroendocrine axes is also adversely affected, namely the hypothalamic-pituitary-adrenal axis and the hypothalamic-pituitary-gonadal axis. This results in an upsurge in the secretion of catabolic hormones like cortisol, coupled with modifications in the secretion of anabolic hormones such as testosterone and insulin-like growth factor 1. It has been postulated that these hormonal shifts may obstruct protein synthesis and enhance proteolysis, thereby compromising muscle recovery processes consequent to intense physical activity [76,83].

During the transition from wakefulness to sleep, there is a shift in autonomic balance towards parasympathetic dominance. Consequently, sleep is associated with a decrease in both sympathetic activity and catecholamines levels, whereas sleep loss is associated with an increase in these variables. Sleep deprivation has also been shown to negatively impact the responsiveness of adrenocorticotropic hormone, adrenaline, noradrenaline, and serotonin receptor sensitivity. Over time, this may lead to altered stress system responsiveness, akin to what is observed in mood disorders [3,14,84,85].

## 4. Physical Activity, Hormones, and Mood Interconnections

Physical activity has been demonstrated to elicit biological responses from various endocrine glands that determine the modulation of hormones’ release and action. Growth hormone, thyroxine, cortisol, reproductive hormones, and other hormones are involved in mood fluctuations. Endocrine effects can alter the receptor density on target neurons, or they can influence neurotransmitter synthesis, metabolism, and release [80,86,87].

### 4.1. The Thyroid Hormones

The interplay between mood disorders and thyroid disorders has been attributed to the influence of thyroid hormones on adrenergic receptor synthesis. Elevated levels of thyroid hormones may increase the adrenergic “tone” in the brain, given the presence of β-adrenergic receptors at this level, as postsynaptic receptors for noradrenaline. Physical activity can lead to an increase in free T4 levels, possibly due to a higher concentration of free fatty acids in the plasma, which can displace T4 from its binding protein. Although T4 is an active hormone, there is no indication that the increase in free hormone levels serves any physiological purpose, and increased TSH levels and activation of the releasing axis would be expected instead. Hormonal changes during physical activity can be attributed to the higher energy demands of an active body. The consistently elevated levels of free T4 in exercising individuals may enhance the arousal feeling caused by noradrenergic brain activity [88,89,90,91].

### 4.2. The Adrenocorticotropic Hormone, Cortisol, and Growth Hormone

The adrenocorticotropic hormone (ACTH) stimulates the adrenal cortex to secrete cortisol, which is released concurrently with β-endorphin (βE) in response to stress events. ACTH, like β-endorphin, is derived from the large precursor protein proopiomelanocortin (POMC), and while ACTH stimulates the hormonal stress system, βE counter-regulates it due to its calming effect. Cortisol receptors are located in various brain areas and are linked to mood and behavior.

Cortisol secretion is triggered in response to physiological stress, physical exercise having a substantial impact on plasma cortisol levels. During exercise, activation of the sympathetic system occurs to stimulate the release of the adrenocorticotropic hormone, which subsequently releases cortisol into the bloodstream. Prolonged aerobic exercise, particularly at elevated intensities, leads to a significant rise in cortisol concentrations compared to exercise of similar duration and intensity in resistance training. Various factors such as age, gender, physical fitness level, exercise intensity, and modality contribute to an individualized pattern of cortisol production. Elevated cortisol levels serve as an indicator of muscle catabolism, heightening the risk of lean muscle tissue loss, particularly concerning for the expanding elderly population. The rate of cortisol production undergoes age-related changes, exhibiting distinct responses to exercise intensities between males and females. Cortisol production correlates with both exercise intensity and duration; however, the increase is not uniform across all exercise intensities. Higher exercise intensities and prolonged durations emerge as primary factors influencing cortisol production, consequently amplifying the potential for muscle catabolism and subsequent muscle loss [68,76,83,92]. In addition to physical activity, sleep is the other major regulatory instance in cortisol synthesis. In contemporary social and work settings, acute sleep deprivation and circadian misalignment are prevalent. Recent discoveries indicate contrasting effects of acute total sleep deprivation and chronic circadian misalignment on cortisol levels. A single night of total sleep deprivation leads to elevated cortisol levels, particularly in the early evening and early morning, while weeks of circadian misalignment result in decreased cortisol levels throughout the 24 h day. Stress ratings rise during the day following acute sleep deprivation, whereas chronic circadian misalignment does not significantly alter stress ratings compared to a synchronized control group. Weeks of circadian misalignment amplify levels of the anti-inflammatory cytokine interleukine-10 (IL-10) and the pro-inflammatory proteins tumor necrosis factor alpha (TNF-α) and C-reactive protein (CRP), particularly during scheduled wakefulness. The TNF-α/IL-10 cytokine balance ratio registers minimal changes during circadian misalignment. Collectively, these findings suggest that acute sleep deprivation, coupled with circadian misalignment, poses a distinct physiological challenge compared to chronic circadian misalignment. Acute sleep deprivation triggers a stress/metabolic response marked by elevated cortisol levels, particularly at night, while chronic circadian misalignment induces a physiological adaptation that reduces 24 h cortisol levels alongside increased pro- and anti-inflammatory proteins [93,94].

The rhythms of growth hormone (GH) and cortisol are significantly affected by sleep characteristics, which are in turn influenced by physical activity. Their levels exhibit a marked surge in response to sufficiently intense exercise, requiring several hours for recovery to baseline levels. Despite their substantial elevation during exercise, these hormones assume a secondary regulatory role in glucose and fat metabolism, with catecholamines and insulin taking precedence. Nevertheless, these hormones are considered crucial not only during exercise but also in the postexercise period. GH release follows a circadian pulsatile pattern and displays peak values nocturnally. Although closely associated with slow-wave sleep, maximal GH levels persist during wakefulness at night. Beyond diurnal rhythms, factors influencing GH secretion at rest and during exercise encompass age, gender, nutrition and sleep patterns, body composition and fitness level, and sex steroid hormones [95,96,97]. Both continuous and intermittent exercise induce an increase in GH concentrations, occurring after the first 15–20 min of activity. The peak GH response to exercise may be tempered by a high-fat pre-exercise meal, while a high-glucose meal exerts a comparatively lesser effect on the GH response. These findings suggest that the composition of the pre-exercise meal can modulate the hormonal response to exercise, potentially influencing the protein-anabolic and lipolytic effects of exercise [97,98]. In addition to metabolic and endocrine modulatory effects, cortisol and GH can display significant behavioral outputs: mood and perceived alertness can impact the ability to engage in physically demanding work and may thus be crucial for human performance. Circadian fluctuations in emotional states may also affect team cohesion in workgroups that require collaboration and communication, such as coaching and team sports [96,99].

### 4.3. Insulin

Physical activity also impacts insulin levels, as plasma insulin levels decrease for at least a day following a single workout. This decline in insulin levels is primarily due to the increase in plasma-free fatty acids, which leads to a faster rate of lipolysis and the release of inhibition of hormone-sensitive lipase in the adipose tissue. This increase in free fatty acids plasma concentration also results in a rise in free fractions of T4, steroid hormones, and the amino acid tryptophan, the precursor of serotonin. Lower glucose levels have been linked to contrary emotional states, but glucose levels in the brain during physical activity are sufficient to maintain its function, suggesting minimal impact on mood during or after physical activity [100,101,102,103,104].

### 4.4. Leptin

The adipose tissue-derived hormone, leptin, is another endogenous molecule highly impacted by physical activity. As a consequence of the fat mass reduction, leptin levels may drop in individuals who practice sports. Acute sessions of moderate physical activity do not profoundly affect the mean leptin plasma levels, considering also the frequent individual variations. A more noticeable reduction could only occur during an activity as demanding as a marathon. Leptin levels may also vary throughout the day, similar to other hormones, which could affect the results obtained after exercise. Neuropeptide Y (NPY), a common peptide neurotransmitter, is inhibited by leptin, accordingly regulating food consumption patterns. Leptin and anxiety may be related, even though the NPY-Y1 receptor and the food-regulatory receptor appear to function in quite distinct ways [82,105,106,107,108,109,110].

## 5. The Interchange between the Beneficial and Detrimental Roles of Reactive Oxygen and Nitrogen Species

Over the course of unceasing evolutionary processes, organisms have intended to develop a diverse array of protective systems aimed at managing the excessive presence of ROS, culminating in what is known as oxidative stress. This terminology pertains to heightened intracellular ROS levels, known to inflict damage upon lipids, proteins, and DNA, a phenomenon long associated with a multitude of pathologies observed in the human context [8,111,112,113,114,115].

**Figure 3:**
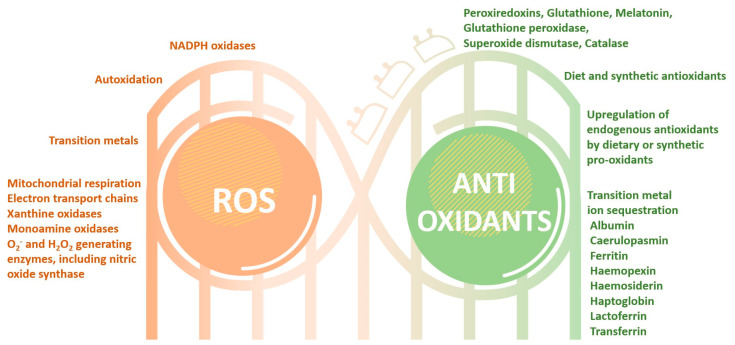
The physiologic continuous process of reactive oxygen species (ROS) generation is counterbalanced by a plethora of antioxidant enzymatic and non-enzymatic mechanisms, whose modulation signals are finely orchestrated and intimately interconnected.

The mechanisms responsible for ROS generation (e.g., via processes such as aerobic respiration or the action of flavin-containing oxidases) and their subsequent rapid elimination (e.g., catalyzed by enzymes like catalase) are universally present in nearly all cell types within organisms, physiologically functioning under a permanent dynamic equilibrium. This self-adjusted balance is often disturbed by the overproduction of oxidative species linked to pathological processes and the presence of diverse xenobiotics, such as pollutants or drugs metabolites, and nevertheless by the insufficient synthesis and activity of the protective systems. The consequences of oxidative stress are contingent upon the degree of damage inflicted by ROS within the cell and the subsequent cellular response to this damage. If the cell proves incapable of rectifying the damage and restoring its functionality or if both exogenous and endogenous antioxidant defenses fail to counteract the ROS-induced harm, the cell may be fundamentally and irreversibly damaged [1,116] (Figure 3).

Nonetheless, it is noteworthy that ROS have also been revealed to function as secondary messengers, transducing extracellular signals to elicit specific cellular responses. For instance, at physiological nanomolar concentrations, hydrogen peroxide emerges as the principal signaling moiety, modulating specific protein targets pivotal in metabolic homeostasis and stress response cascades. These orchestrated events facilitate cellular adaptability to dynamic environmental shifts and stressors. Furthermore, an array of additional reactive species, including nitric oxide, hydrogen sulfide, and oxidized lipids, participate in redox signaling phenomena. An affluence of scientific investigations has significantly contributed to the development of the concepts related to oxidative stress and the elucidation of the complex mechanisms governing ROS production and regulation, as well as their integral involvement in processes underpinning cellular signaling [116,117].

### 5.1. Reactive Oxygen and Nitrogen Species’ Impact on Sustaining the Biological Systems’ Homeostasis

Reactive oxygen species constitute a class of highly reactive molecules encompassing radicals such as O_2_• and OH•, as well as non-radical oxygen derivatives like H_2_O_2_. These molecules serve pivotal roles in cellular signaling and are indispensable for upholding the biological systems’ homeostasis. Additional ROS are generated through interactions of O_2_• with other molecules, leading to hydrogen peroxides and hydroxyl radicals, which interconvert with reactive nitrogen species (RNS) exhibiting comparable effects to ROS. The primary source of ROS is commonly attributed to inefficient electron transfer within the mitochondrial respiratory chain, although various enzymatic and non-enzymatic mechanisms can also give rise to ROS.

Nitrosative stress exhibits a close interrelation with oxidative stress, wherein ROS, including the superoxide anion, singlet oxygen, hydroxyl radical, hydrogen peroxide, peroxynitrite anion, and nitric oxide, actively participate. The peroxynitrite anion (ONOO^−^) generated during oxidative stress can induce nitration of various biomolecules, including proteins, lipids, and DNA, resulting in the formation of 3-nitrotyrosine (3-NT). The hallmark feature of nitrosative stress lies in tyrosine nitration, a post-translational modification of proteins stemming from their interactions with RNS/ROS. Recent investigations underscore the specificity of 3-NT formation as a biomarker for nitrosative stress, offering insights into monitoring intracellular ONOO^−^ production, its localization, and the severity of ensuing cell death [118,119,120].

All oxygen-utilizing cells partake in oxygen metabolism, denoted as cellular respiration, which yields ROS as an obligatory byproduct of aerobic existence. Exogenous metals, redox compound recycling, radiation, chemotherapy, carcinogens (including estrogenic compounds), as well as diverse dietary and environmental factors, have the potential to generate ROS. Elevated ROS concentrations typically trigger nonlinear cellular responses. In both physiological and pathological contexts, there is a delicate equilibrium between oxidant and antioxidant systems, which leads to the continual regulation of ROS production, distribution, and deactivation [101,121].

The vital systems for ROS detoxification encompass a variety of individual or synergistic endogenous antioxidants, including the catalase enzyme family, glutathione, thioredoxin-related derivatives, and superoxide dismutase, as well as exogenous antioxidants such as reduced glutathione, carotenoids, and vitamins C and E. Nonetheless, redox homeostasis can readily become perturbed, often favoring oxidants, thus shifting ROS levels from physiological to potentially detrimental ranges, displaying oxidative and nitrosative stress [115,122].

### 5.2. ROS Over-Production and Effects’ Accumulation Can Exert a Significant Influence on the Pathogenesis and Progression of Chronic Diseases

Oxygen and the entrainment of circadian rhythms are indispensable components in a multitude of physiological processes aimed at preserving homeostasis. These processes span a wide range, encompassing the regulation of blood pressure, the orchestration of sleep/wake cycles, and the intricate modulation of cellular signaling pathways that exert a pivotal influence on both health and the pathogenesis of diseases [2,101].

In instances where the human body or its constituent cells undergo significant stress, their capacity to effectively govern internal systems, comprising the maintenance of redox equilibrium and circadian temporal patterns, may become compromised. This compromise is evident at both the cellular and organism levels, and it can precipitate a cascade of adverse consequences. Among these consequences are the emergence and progression of various diseases, including but not limited to cardiovascular syndromes, neurodegenerative disorders, and cancer [4,10,56,123,124,125,126,127].

**Figure 4:**
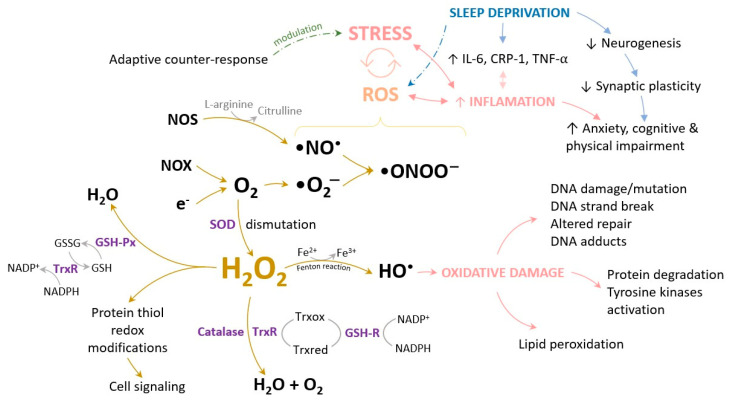
The generation of reactive species is at the same time a physiologic and a pathological process, dictated by the disequilibrium between the amount produced and the extent to which they are neutralized by active antioxidant mechanisms. The signaling processes involving reactive oxygen species (ROS) occupy a pivotal position in the control of proinflammatory mechanisms, protein redox adjustments, cellular proliferation, and apoptotic cell death. The safeguarding of cellular and tissue integrity against the detrimental effects of elevated ROS concentrations is effectively mediated by the actions of antioxidant defense enzymatic and non-enzymatic factors. *ROS—reactive oxygen species, IL-6—interleukin 6, CRP-1—C reactive protein, TNF-α—Tumor necrosis factor-alpha, NOS—Nitric oxide synthase, NOX—NADPH oxidases, SOD—Superoxide dismutase, GSSG—oxidized glutathione, GSH—reduced glutathione, GSH—Px-Glutathione peroxidase, TrxR—Thioredoxin reductase, GSH-R—Glutathione reductase.*

An increase in ROS levels can be incited by a plethora of factors, encompassing sedentary behavior, sleep deprivation, consumption of processed dietary items, exposure to diverse chemical agents, the ingestion of alcohol and toxic substances, and the exaggerated use of drugs on long periods of time [14,17,128,129,130]. Disruption in the delicate equilibrium between the robust antioxidant defense system and ROS production can exert a significant influence on the pathogenesis of diabetes, metabolic syndrome, myocardial infarction and stroke, sleep disorders, cognitive and physical impairment, inflammatory states, and DNA damage connected malignancies [43,51,55,56,130] (Figure 4).

These defense mechanisms, primarily rooted in antioxidant protection, involve an array of strategies, encompassing both enzymatic and non-enzymatic constituents. Among the prominent non-enzymatic antioxidants are glutathione (GSH), vitamins—particularly vitamin C and E, selenium, carotenoids, thioredoxins (Trx), lipoic acid (ALA), and flavonoids [12,131,132]. In parallel, the presence and activity of cellular antioxidant enzymes serve as the fundamental defense mechanism against ROS, with the most notable ones being superoxide dismutase (SOD), catalase (CAT), glutathione peroxidase (GSH-Px), reduced-to-oxidized glutathione ratio, and glutathione transferase (GST). Their primary mission revolves around preventing oxidative tissue damage induced by oxygen radicals. Their responsibility covers both the prevention of ROS formation and the subsequent neutralization of ROS into inactive compounds [1,133].

## 6. Physical Exercise as a Key Element of the Oxygen Fate in the Human Organism

Oxidative stress is a well-known consequence of physical exercise, occurring even after a single sport session, and the generation of free radicals is considered a key indicator of the muscular and systemic responses to physical activity. This inference is based on scientific evidence from exercise studies conducted in the last decades. Exercise increases the oxygen demand, especially in skeletal muscle, which alters the blood distribution to different organs. Moreover, exercise-induced muscle injury attracts neutrophils and macrophages to the site of damage. These physiological changes induced by acute exercise enhance the production of free radicals and cause oxidative modifications to biomolecules. Recent advances in biochemical and molecular techniques have revealed that free radicals are also involved in some of the physiological adaptations that occur after exercise training. Therefore, it can be argued that the physiological effects of exercise-induced free radicals are both beneficial and detrimental, depending on the exposure period and intensity of the training, the basis of a hormetic factor [52,134,135].

Hormesis is characterized as a phenomenon wherein exposure to a low dose of a chemical agent or environmental factor, which may be harmful at higher doses, elicits an adaptive beneficial effect on the cell or organism. This concept embodies the fundamental principles of “conditioning” and “adaptation” [136,137,138]. Conditioning and adaptation, often treated as synonymous and used interchangeably, describe the idea that modest levels of stress activate or upregulate existing cellular and molecular pathways. This activation enhances the ability of cells and organisms to withstand more substantial stressors [139]. The concept of hormesis underlies our understanding of how exercise conditions the body and induces long-term adaptation: moderate physical activity is associated with reduced risk of illness and mortality, whereas excessive activity elevates these risks [138,140,141,142].

During exercise, the body encounters various homeostatic perturbations, including thermal, metabolic, hypoxic, oxidative, and mechanical stress. Antioxidant supplements and non-steroidal anti-inflammatory drugs (NSAIDs) may aid in preserving or enhancing muscle adaptations in older individuals with compromised antioxidant defense systems or chronic low-grade inflammation [143]. Conversely, these interventions in younger individuals may attenuate exercise-induced increases in insulin sensitivity and muscle protein synthesis. Therefore, the outcomes of such interventions may vary among different exercising populations [144,145].

Exploring the continuum of hormesis, interest has grown in applying stress to skeletal muscle in different time frames of the exercise to stimulate a greater adaptation cascade of the body. This stress can be induced by limiting carbohydrate intake, blood flow restriction through low-intensity isometric or eccentric contractions (mechanical “preloading”), cryotherapy, or heat stress [146].

### 6.1. The Signaling Pathways Embodied in Exercise-Induced Oxidative Stress

Endurance exercise training induces the generation of oxidants in skeletal muscles and activates the enzymatic antioxidant mechanisms, upregulating the expression and activity of antioxidant enzymes SOD1, SOD2, GSH-Px, and CAT in skeletal muscle. Endurance exercise training has been shown to enhance the total SOD activity in oxidative type I (*soleus*) and IIa (*red gastrocnemius*) skeletal muscle fibers. The duration and consistency of endurance training also influence the increase in both cytosolic and mitochondrial GSH-Px activity in oxidative skeletal muscle (type I and IIa) fibers. Moreover, endurance training augments the CAT activity at the level of peroxisomes and mitochondria in highly oxidative muscles [15,52,147].

Exercise-induced oxidative stress and the mitochondrial biogenesis cascade are triggered by endurance exercise training, which involves repeated muscle contractions. It is well known that newly formed mitochondria are highly efficient and produce fewer ROS for a given amount of adenosine triphosphate (ATP). Proteins involved in mitochondrial biogenesis such as transcriptional co-activator peroxisome proliferator-activated receptor γ co-activator 1α (PGC-1α), nuclear respiratory factor 1 (NRF-1), and mitochondrial transcription factor A upregulate their expression due to consistent exercise training. The mitochondria are not the only sources of ROS during muscular contraction: superoxide activity in the cytosol is amplified by muscle contraction, followed by a delayed increase in the mitochondria, and nicotinamide adenine dinucleotide phosphate-oxidases (NADPH oxidases, NOX) have also been identified as potential causes of superoxide production. In this regard, it has previously been shown that ROS generation increases in isolated mitochondria after an acute muscular contraction compared to a relaxed skeletal muscle sample [73,148].

Moderate ROS levels are critical in the signaling pathways of mitochondrial biogenesis; therefore, decreased ROS-stimulated mitochondrial biogenesis is correlated to a diminished PGC-1α expression. Vitamin C and other antioxidant molecules (vitamin E, A, polyphenols) supplementation reduces ROS levels and prevents enzymatic antioxidant activity. Additionally, several scientific findings support the idea that a reduced PGC1α expression is linked to a decreased activity of all antioxidant enzymes, which is the first endogenous antioxidant barrier [143,147].

Muscle fatigue emerges from damaged muscle fibers triggered by ROS generation. However, it has become increasingly evident that a minor biological signal, such as a modest amount of ROS, might induce the transcription of important genes implicated in the antioxidant defense system. Muscle fatigue and oxidative damage are positively correlated, which may be a key strategy for dietary therapies to improve exercise performance. Taking into account the reactive oxygen species scavenging effects instrumenting a limitation in the muscle damage occurring due to intense exercise, antioxidants supplementation could be an effective approach for people practicing extended sport sessions [147,149,150].

### 6.2. The Role of Antioxidant Molecules’ Supplementation in Sports Training

One of the detrimental outcomes of increased reactive oxygen species (ROS) levels is the impairment of muscle function, different histological changes, and muscular pain, all leading to reduced athletic performance. To mitigate these negative consequences, nonenzymatic antioxidants as supplements or from dietary sources have been assessed [44,54,115,132,135,143,149,151,152,153,154,155]. The efficacy of integrating nonenzymatic antioxidant supplements in the strategy of enhancing human resistance exercise performance by reducing the negative impact of ROS during exercise has been extensively surveyed. Preclinical investigations and some recent human studies have yielded conflicting findings on the direct effects of incorporating non-enzymatic antioxidants during resistance training, which deviates from the original concept of protection [115,135,143,149]. Several studies have demonstrated that physical exercise can lead to an increase in free radical production and a decrease in antioxidant enzyme levels in different tissues and organs, ultimately leading to oxidative stress. Nevertheless, the magnitude of biological stress is contingent on the type and intensity of exercise performed. As the production of free radicals increases, so does oxygen consumption and oxidative phosphorylation.

Moreover, the body can adapt to the typical, moderate generation of reactive oxygen species (ROS) during exercise, which enables more efficient enzymatic removal and consequently enhances the mitochondrial oxidative capacity and efficiency, leading to the production of new, more efficient mitochondria, this adaptation preventing the harmful overproduction of ROS.

The impact of antioxidants supplementation on oxidative stress induced by exercise remains a controversial topic. The effectiveness of the antioxidants may depend on specific training types and scenarios, such as different sport types (e.g., aerobic vs. anaerobic) or particular training timing, being necessary to carefully select and describe the appropriate training stimuli and monitor the athlete throughout the entire training process [149,151,154].

In recent years, numerous scientific studies have brought attention to a potential correlation between oxidative stress and a series of bioactive compounds present in plants [50,54,135,155]. Consequently, the focus has shifted towards investigating the effects of biological compounds, such as polyphenols, found both in common foods and in nutraceuticals.

Polyphenols are abundant in the human diet, with Western populations typically consuming approximately 1–2 g of polyphenols daily. Food sources of polyphenols include fruits and vegetables, green and black tea, red wine, coffee, chocolate, and extra virgin olive oil, with berries, apples, grapes, pears, and other similar fruits typically having high polyphenol content (200–300 mg per 100 g). Polyphenols are antioxidants, which makes them a viable nutritional supplement to counteract the oxidative stress induced by physical activity, and additionally, they possess several other biological activities. At the moment, different design, timing, and dosing approaches have been used to study the effects of various polyphenols in a broad spectrum of operational settings [135,156,157,158,159,160].

In addition, recent studies have revealed that the supplementation of L-citrulline with GSH during resistance training increases lean mass in resistance-trained males compared to placebo, owing to the synergistic mechanism of the two molecules. L-citrulline, a nonessential amino acid, is co-produced with nitric oxide (NO) as an end-product of nitric oxide synthase (NOS). Unlike L-arginine, L-citrulline can bypass hepatic metabolism and is transported to the kidneys, where it is directly converted into L-arginine. This makes exogenous L-citrulline supplementation a reliable alternative to increase the amount of L-arginine available to NOS. Unlike L-arginine, L-citrulline faces limited intestinal catabolism, as it is not metabolized by arginases, and arginosuccinate synthase activity is low in enterocytes. Low molecular weight thiols like glutathione (GSH) have demonstrated the ability to up-regulate the NO pathway, forming S-nitrosoglutathione (GSNO) upon interaction with NO. GSH acts as an NO donor, facilitating NO transfer reactions. It stabilizes low levels of NO and gradually releases NO through GSNO conversion. Additionally, GSH reacts with newly created free radicals, safeguarding NO from oxidative damage and enhancing its effectiveness [161,162].

Dietary support has the potential to partially ameliorate exercise-induced alterations without impeding the requisite signaling processes crucial for training adaptations. Athletes are advised to refrain from consuming foods that may amplify oxidative stress while increasing their intake of foods rich in antioxidants can elevate plasma antioxidant levels, affording protection against numerous chronic complaints. The optimization of nutrition, when combined with exercise, is firmly established as an effective ergogenic practice for enhancing athletic performance. Nutraceutical compounds are typically derived from diverse sources, including foods, plants, and even microorganisms. In recent years, the nutraceutical industry has transitioned from being a conceptual area within biomedical research to evolving into a value-added segment with promising prospects [156,159,162].

## 7. The Path Forward in Aligning Nutrition, Timing, and Physical Performance

Considering the importance of exercise from moderate to more intensive approaches, from sustained physical training to athletic performance, a series of coherent guidelines have been detailed lately, in order to augment the adherence of people to a series of lifestyle principles meant to expand the quality and span of their existence. The International Society of Sports Nutrition has evaluated existing literature on energy drinks, including caffeine consumption, and the best timing for macronutrient intake in relation to exercise performance and body composition for healthy adults, particularly those who are highly trained in the context of exercise performance and provided some important insights. Caffeine supplementation has shown consistent enhancements in various aspects of exercise performance, although results can vary among studies. These benefits, typically of moderate magnitude, encompass improvements in muscular endurance, movement speed, muscular strength, sprinting, jumping, throwing performance, and various sport-specific activities, both aerobic and anaerobic.

### 7.1. The Implications of Caffeine Intake in Physical Training

Aerobic endurance exercises seem to consistently benefit the most from caffeine intake, with moderate to substantial improvements observed. Effective doses of caffeine for performance enhancement typically range from 3 to 6 mg per kilogram of body mass, although there may be a lower threshold of effectiveness at around 2 mg/kg. Extremely high doses, such as 9 mg/kg, are associated with a higher risk of side effects and are not necessary to achieve performance benefits. The timing of caffeine supplementation is commonly around 60 min before exercise, although the optimal timing can depend on the caffeine source. For example, caffeine from chewing gum may require a shorter waiting period before exercise initiation compared to caffeine capsules [163].

Caffeine’s performance-enhancing effects extend to both trained and untrained persons. Individual differences in sport and exercise performance, as well as potential side effects like disrupted sleep or increased anxiety following caffeine consumption, may be influenced by genetic variations related to caffeine metabolism and individual physiological and psychological responses. Caffeine has been found to have an ergogenic effect on cognitive function, including improvements in attention and vigilance, in most individuals. Under conditions of sleep deprivation, caffeine may enhance both cognitive and physical performance in some people. Caffeine can be beneficial when combined with endurance exercise in hot conditions and at high altitudes, with effective doses ranging from 3 to 6 mg/kg and 4 to 6 mg/kg, respectively. Energy drinks and pre-workout supplements containing caffeine have been shown to enhance both anaerobic and aerobic performance [23,163,164,165,166,167].

### 7.2. Amino Acids and Carbohydrates Optimal Consumption Related to Physical Exercise

Nutrient timing involves strategic planning and consumption of whole foods, fortified products, and dietary supplements with the aim of enhancing recovery, tissue repair, stimulating muscle protein synthesis, and improving mood states after intense exercise. In addition to maintaining a well-balanced diet, individuals seek ergogenic aids that possess potential direct or indirect effects to enhance physical performance for better adaptation to physical training. Among ergogenic products, branched-chain amino acids (BCAA)—specifically valine, leucine, and isoleucine—hold widespread popularity, as their intake, coupled with resistance exercise, promotes muscle protein synthesis. However, the evidence supporting the standalone efficacy of BCAA for inducing muscle hypertrophy in humans is somewhat inconclusive [168,169].

In healthy active subjects, the dynamic balance between protein degradation and synthesis governs the maintenance of skeletal muscle protein homeostasis. During fasting, muscle protein breakdown surpasses synthesis, resulting in protein loss. Conversely, in the postprandial state, synthesis outpaces degradation, fueled by nutrient intake, especially proteins and carbohydrates, which stimulate muscle protein synthesis and insulin release, suppressing degradation. In adults, physical exercise and nutrient availability are the primary drivers of muscle protein synthesis, accelerating the incorporation of amino acids obtained through the diet into skeletal muscle proteins. The optimal and prolonged stimulation of muscle protein synthesis occurs when physical exercise, particularly resistance exercise, is combined with protein intake, due to increased tissue sensitivity to the anabolic properties of amino acids, sustaining the stimulation for approximately 24 h post-workout [168,170].

During resistance exercise, carbohydrate ingestion helps maintain stable blood glucose levels and increases muscle glycogen stores. So, in order to maximize glycogen supplies, a high-carbohydrate diet is recommended, as these stores are most depleted by high-volume exercise. Consuming carbohydrates alone or with protein during resistance exercise improves glycogen storage, reduces muscle damage, and enhances short- and long-term training adaptations. Meeting daily protein intake, distributed evenly across multiple protein feedings, is crucial for physically active individuals. The size and timing of pre-exercise meals may influence the need for post-exercise protein intake. Immediate post-exercise protein consumption, in the first two hours, from high-quality sources effectively stimulates muscle protein synthesis [171].

## 8. Promising Research Directions, Future Clinical Implications, and Possible Translations into Preventive or Therapeutic Strategies

The high scientific interest expressed by the numerous and complex clinical and preclinical studies on chronobiology acknowledge its importance in maintaining a balanced state of health, as it connects the dots between nutrition, cognitive and physical performance, the incidence of particular diseases, hormonal balance, sleep homeostasis, and above all, timing, all demonstrated in observational and prospective approaches. The slight difference between time and timing is exactly the synchronization in its most profound understanding of all actions and activities employed by a human being with the inner biological environment, depicted by various biochemical arrangements: from the complete array of hormonal synthesis to the expression of circadian genes, the enzymatic modulation of metabolic pathways, the cellular signaling orchestrated by several key molecules.

We must decipher the bidirectional interdependence between the human organism and the environment and give it a deep sense of meaning through the detailed roles studied continuously in order to comprehend their mechanisms of action and the regulation feedback. Applying chronobiological principles has proved its importance in pathology, mainly for patients at risk due to their shift work schedules, intensive traveling across different time zones or sleep disorders; in chronotherapy, as the prescription of a series of therapeutic agents has tremendously changed the outcome of their administration when aligned to certain time tables or to the patient chronotype, considering an individualized therapeutic approach; in chrononutrition, as eating patterns and fasting frames are a pertinent way to limit a series of metabolic disorders in vulnerable patients, or a perfect approach in enhancing the organism performance in routine or special physical or psychological activities.

The present challenge is to identify new *zeitgebers* able to entrain the human circadian clock and to use them for increasing the positive impact of daily routines on the most unexpected processes that conduct to the homeostatic state of a cell, tissue, organ, system, or apparatus, and in the end, at the highest state of physiological equilibrium for the entire organism. Physical exercise might embody one such *zeitgeber* given its ability to entrain the human circadian system. We aimed to detail the characteristics required to modulate the crosstalk between the molecular clock and redox biology, revealing the pertinent mechanistic pathways such as those that control the antioxidant defense and DNA protection and repair.

Rather than admitting there is an optimum time in exercise responses and body adaptations to effort and energy expenditure, we comprehensively identified the distinct differences dependent on the individual’s health state, chronotype, genes, personal history and exposure to effort, fitness status, labor and domestic paradigms and preferences, including mealtimes and dietary patterns, concluding that physical training recommendation should be unquestionably personalized.

## 9. Conclusions

Recent advancements in the field of molecular biology connect circadian rhythms with the redox environment dynamics, especially in the view of advocating or scrutinizing physical activity for health. In contemporary understanding, redox reactions are increasingly acknowledged as a fundamental component of the cell signaling machinery, operating alongside other well-established biochemical processes that finely regulate human metabolism. In light of this comprehensive review, it becomes evident that certain nutrients, used in appropriate ratios and following particular schemes can have antioxidant, stamina, and sleep modulating properties, acknowledging the key role of nutritional interventions tailored to the age and gender group. Additionally, personalized exercise adaptations designed to enhance homeostatic mechanisms, thereby promoting overall health, sleep quality, energy expending, and exercise adaptations in both the general and athletic populations, certificate further exploration as efficient tools in adjusting the quality of life and physical performance. Beyond the role of light, physical activity plays a crucial part in entraining environmental oscillators via the master biological clock. This initiation triggers a cascade of physiological functions involving hormones, enzymes, and neurotransmitters, forming a complex network governed by multiple feedback loops.

Considering the fact that the adaptability of an organism is heavily contingent on its internal biological clock, which is influenced by environmental factors, usual clinical interventions should deeply explore the circadian pattern of each patient, characterize the individual oxidative pattern, include the nutritional and sleep particularities, address the physical training level, and corroborate age and gender characteristics in the routinely driven interventions. All evidence indicates that sleep quality, chrononutritional interventions, time-framed physical activity, and intrinsic circadian rhythms exert significant control over the functionality of the entire human body, ensuring the state of homeostasis.
